# Case Report: Esophagectomy and Azygos Continuation of the Inferior Vena Cava: A Lethal Combination

**DOI:** 10.3389/fcvm.2022.780646

**Published:** 2022-03-31

**Authors:** Yan Zhang, Zheng Ding, Teng Mu, Xue Pan, Guoqing Zhang, Xiangnan Li

**Affiliations:** ^1^Department of Thoracic Surgery and Lung Transplantation, The First Affiliated Hospital of Zhengzhou University, Zhengzhou, China; ^2^School of Nursing and Health, Zhengzhou University, Zhengzhou, China

**Keywords:** esophageal carcinoma, esophagectomy, azygos continuation, congenital inferior vena cava variant, artificial vascular bypass

## Abstract

Azygos continuation of the inferior vena cava (IVC) is rare in the general population and even rarer among patients with esophageal carcinoma. In 90% of cases, this congenital IVC variant is isolated and does not affect the patient’s growth or development. However, thoracic surgery such as esophagectomy would be fatal if the flow through this connection was interrupted. We present a case of minimally invasive esophagectomy in a patient with azygos continuation of the IVC.

## Introduction

Azygos continuation of the IVC is rare in the general population. In a large series of 55,457 pregnant women who underwent prenatal examinations, an incidence of 0.02% (11/55457) was determined for azygos continuation of the IVC. Most of these cases (90%) were not associated with other anatomical variants (1). Azygos continuation of the IVC is considerably rarer in patients with esophageal carcinoma, and only six cases have been reported (2–4). Awareness of this type of combined condition is crucial in esophagectomy, and it would be fatal if the azygos vein was divided while following the usual procedure. Here, we report a case of minimally invasive esophagectomy in a patient with azygos continuation of the IVC. Written informed consent was obtained from the patients’ legal guardian for the publication of any potentially identifiable images or data included in this article.

## Case Presentation

A 61-year-old female had dysphagia, and a lower thoracic squamous cell carcinoma (SCC) was detected *via* an upper gastrointestinal endoscopy (distance from incisor tooth, 30–35 cm) at a local hospital. Potential metastasis was ruled out by chest and abdomen CT scan, brain MRI and bone scintigraphy. The physical examination and laboratory tests were unremarkable. A cT3N0M0 primary SCC of the esophagus was diagnosed, and a minimally invasive McKeown esophagogastrectomy was performed on March 28, 2021, without a detailed preoperative evaluation for vascular malformation.

During the surgery, the arch of the azygos vein was unusually enlarged and was unfortunately routinely divided without recognizing an azygos continuation of the IVC. The patient developed progressive hypotension and oliguria after 60 min, and repeated doses of dopamine and ephedrine did not improve this deterioration. The esophagogastrectomy was completed after the use of a large amount of vasopressors to maintain the patient’s blood pressure. Unfortunately, the patient’s condition progressively deteriorated.

**FIGURE 1:**
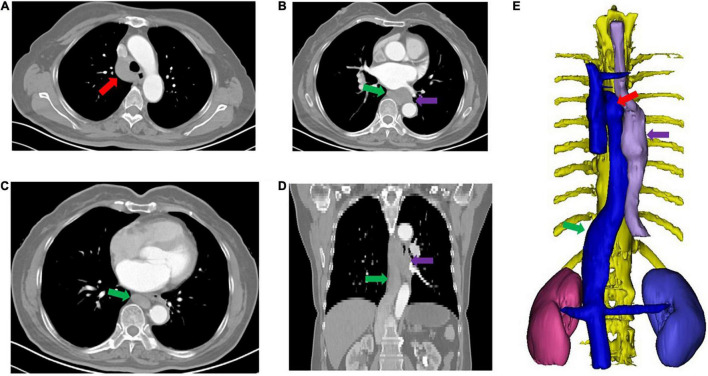
CT reconstruction showing azygos continuation of the inferior vena cava (not preoperatively recognized). **(A–D)** Show the enlarged arch of the azygos vein and azygos vein. **(E)** Shows hepatic segment absence of the inferior vena cava with azygos continuation. The red arrow shows the enlarged arch of the azygos vein; the green arrow shows the azygos vein; the purple arrow shows the tumor.

The final diagnosis was obtained after reviewing the patient’s preoperative CT scan. The absence of the hepatic segment of the IVC was detected, and the 3D CT reconstruction showed an azygos continuation of the IVC (Figure 1). Then, the patient was transferred to our tertiary hospital for further treatment.

**FIGURE 2:**
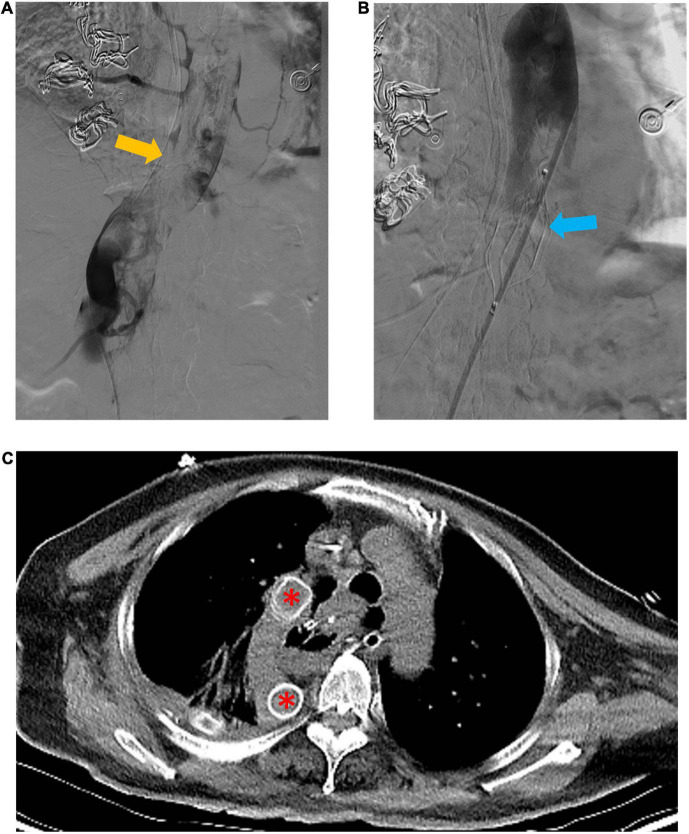
Emergency digital subtraction angiography (DSA) of the inferior vena cava and artificial vascular bypass to reconstruct venous drainage. **(A)** Shows a large thrombus (yellow arrow) in the azygos vein. **(B)** Shows the inferior vena cava filter (blue arrow) superior to the bilateral renal vein. **(C)** Shows the well-located artificial vessel (red asterisk).

An emergency digital subtraction angiography (DSA) of the inferior vena cava and double renal vein was performed 24 h post-operation. Angiography demonstrated a large thrombus at the azygos vein from the level of the diaphragm to the level where it was divided during surgery (Figure 2A). The patient underwent a thrombectomy and the placement of an inferior vena cava filter above the bilateral renal vein (Figure 2B). Artificial vascular bypass grafting was used to further reconstruct the venous drainage: the proximal part was anastomosed to the right atrium, and the distal end of the graft was anastomosed to the azygos vein at the level where it was divided during surgery. The artificial vessel was easily located on the postoperative routine CT evaluation (Figure 2C) (April 3, 2021). Unfortunately, the patient remained anuric after surgery, and continuous renal replacement therapy (CRRT) was used to provide renal support.

**FIGURE 3:**
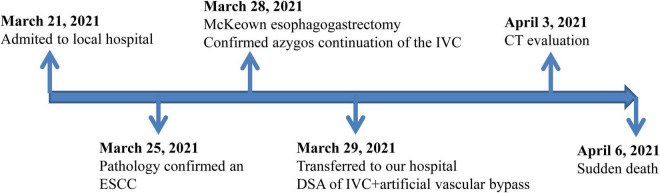
Timeline of events of the patient from admission to discharge.

On April 6, 2021, the patient suffered from sudden cardiac arrest and died despite emergency rescue efforts. A pulmonary embolism was deemed the most likely cause of sudden death according to dilation of the pulmonary artery detected by bedside ultrasound (Figure 3). Unfortunately, effective management such as thrombolytic therapy and hemodynamic support was not initiated because of failure of rescue.

## Discussion

Embryogenesis of the IVC (between the 6th and 8th weeks of embryonic life) is a complex process, and it involves the formation of several anastomoses between several embryonic veins. In general, the IVC is composed of four segments: a hepatic, a suprarenal, a renal, and an infrarenal segment. Failure to form the right subcardinal-hepatic anastomosis is thought to be the embryonic event of azygos continuation of the IVC. Consequently, blood is shunted from the suprasubcardinal anastomosis through the retrocrural azygos vein.

This malformation does not affect the patient’s growth and development in most cases. However, it would be fatal if the patient suffered from diseases that needed to be treated with surgical intervention, such as a right transthoracic esophagectomy (3, 5). Considering the unremarkable physical examination and laboratory test results of patients with such an anomaly, recognition of the warning signs (enlarged azygos vein and arch of the azygos vein) of this anomaly as visualized on preoperative CT and during intraoperative exploration is vital. To date, there have been only six reported cases of azygos continuation of the IVC in patients undergoing esophagectomy (2–4, 6) (Table 1). Three patients were safely discharged with adequate arch of azygos vein protection (3, 4). Unfortunately, the arches of the azygos vein in the other three patients were routinely divided: one patient died within 15 h after esophagectomy (3), one patient died within 1 day after esophagectomy (5) and one survived after experiencing dangerous postoperative complications (acute renal insufficiency, hypotension, and huge azygos vein thrombus) (2). Similarly, there is also a report of an NSCLC patient who died after ligation of the azygos vein (7). However, we should note that it is likely that the true incidence is much higher, as death cases are less likely to be published.

**TABLE 1:** Reported cases of azygos continuation of the inferior vena cava in patients undergoing esophagectomy.

Author	Country	Age, sex	Operation	Combined malformation	Arch of azygos vein	Discharge status
Bronshtein et al. (1)	Hungary	52 y, male	Open	No	Protected	Alive
Veltman et al. (2)	Spain	62 y, male	Open	No	Divided	Died within 1 day
Wang et al. (3)	China	52 y, male	Open	No	Divided	Died within 15 h
Wang et al. (3)	China	56 y, male	Open	Double IVC	Protected	Alive
Palotás et al. (4)	Netherlands	62 y, female	Open	No	Divided	Alive
Martín-Malagón et al. (5)	Japan	58 y, male	NR	Double IVC	Protected	Alive
Zhang et al.	China	61 y, female	Minimally invasive	No	Divided	Died within 9 days

*IVC, inferior vena cava; y, year.*

Interestingly, the case of a patient reported by Veltman et al. survived even though the enlarged azygos was divided (2). Furthermore, eight patients with interruption or stenotic lesion of the IVC without a well-developed azygos/hemiazygos continuation have been reported (8). In such patients, venous return from the bilateral kidneys and lower extremities occurs exclusively through potential vessel variation or the collateral veins, such as (accessory) hemiazygos venous return, paravertebral collaterals (9) and portocaval communication. However, under circumstances of surgical ligation, compensatory collaterals do not have enough time to develop and are not sufficient for vein return. Similar to the case reported by Martín-Malagón et al. (5), artificial vascular graft was used to reconstruct the damaged arch of azygos vein in our study. Unfortunately, the two patients undergoing artificial vascular bypass died due to thromboembolic events, which may be additional evidence of a hypercoagulable state related to cancer or a cava filter. Scholars have pointed out that immediate reconstruction of the damaged arch of the azygos vein has important clinical importance in preventing graft thrombosis formation (5). Furthermore, active graft thrombosis prophylaxis after reconstruction of the arch of the azygos vein maybe of great clinical importance (10, 11).

We describe a patient who died after undergoing esophagectomy due to the presence of an azygos continuation of the IVC, and we systematically reviewed the limited literature related to this anomaly. The implications of this study for clinical practice are as follows. (1) Any patient who undergoes esophagectomy should have their CT reviewed for macrovascular malformations by the surgical team to prevent complications. Additionally, intraoperative exploration is the last opportunity to recognize an enlarged azygos vein and arch of the azygos vein to prevent fatal complications. (2) In some rare conditions (hemiazygos venous return, and abundant paravertebral collaterals), ligation of the enlarged azygos is not necessarily fatal, and a “wait and see” strategy may be reasonable. (3) In most cases, artificial vascular reconstruction is necessary and may be an appropriate intervention.

## Data Availability Statement

The data that support the findings of this study are available from the corresponding author upon reasonable request.

## Ethics Statement

Written informed consent was obtained from the patients’ legal guardian for the publication of any potentially identifiable images or data included in this article.

## Author Contributions

YZ, ZD, and TM contributed to the conception and design of the study. YZ organized the data, performed the statistical analysis, and wrote the first draft of the manuscript. YZ, XP, GZ, and XL wrote the sections of the manuscript. All authors contributed to manuscript revision, read, and approved the submitted version.

## Conflict of Interest

The authors declare that the research was conducted in the absence of any commercial or financial relationships that could be construed as a potential conflict of interest.

## Publisher’s Note

All claims expressed in this article are solely those of the authors and do not necessarily represent those of their affiliated organizations, or those of the publisher, the editors and the reviewers. Any product that may be evaluated in this article, or claim that may be made by its manufacturer, is not guaranteed or endorsed by the publisher.
